# Novel Functional Indices of Masticatory Muscle Activity

**DOI:** 10.3390/jcm10071440

**Published:** 2021-04-01

**Authors:** Michał Ginszt, Grzegorz Zieliński

**Affiliations:** 1 Department of Rehabilitation and Physiotherapy, Medical University of Lublin, 20-093 Lublin, Poland; 2 Department of Sports Medicine, Medical University of Lublin, 20-093 Lublin, Poland; grzegorz.zielinski@umlub.pl

**Keywords:** electromyography, masticatory muscles, functional indices, temporal muscle, masseter muscle

## Abstract

The aim of the present study was to analyze novel functional indices of masticatory muscle activity and compare them to existing and commonly used indices in patients with temporomandibular disorders (TMDs) and healthy adults. Based on the Research Diagnostic Criteria for Temporomandibular Disorders, 78 adult women qualified for the study. Subjects were divided into two groups: diagnosed TMDs (*n* = 36; mean age: 23.4 ± 2.6 years) and healthy adults (*n* = 42; mean age: 22.4 ± 2.3 years). Measurements of the bioelectric activity of the temporalis anterior (TA), superficial masseter (MM), and anterior bellies of the digastric muscle (DA) were carried out using the BioEMG III ™. Functional Clenching (FCI) and Functional Opening (FOI) indices were obtained as the ratio of the difference between the mean muscle root mean square (RMS) potentials during functional activity, including clenching (CL) and opening (MMO), and mean muscle resting (REST) potentials. Next, based on FCI and FOI indices, the Functional Clenching Activity Index (FCAI), Functional Clenching Symmetry Index (FCSI), and Functional Opening Symmetry Index (FOSI) were obtained. The statistical analysis showed significant differences in activity index left-sided (AcI_L_) and Activity index both-sided (AcI_tot_) between TMDs and healthy women during rest measurements. The significant differences between both groups were noted in terms of all Functional Clenching Indices except Functional Clenching Index for MM right-sided (FCI_MM-R_). In all analyzed FCI indices, the control group showed higher values compared to the TMDs. Moreover, a significant difference between TMDs and controls was observed within Functional Clenching Activity Index left-sided (FCAI_L_) (14.56 vs. −0.45, *p* = 0.01). Both functional indices, and asymmetry (AsI) and activity (AcI) indices seem to be reliable in assessing symmetry and activity within masticatory muscles. Further studies should be performed to verify the effectiveness and suitability of the assessment of masticatory muscles using functional indices.

## 1. Introduction

Surface electromyography (sEMG) is widely applied in dentistry to analyze bioelectric masticatory muscle activity and is commonly practiced in both clinical and research fields [1]. In numerous studies, electromyographic assessment of masticatory muscle activity has been used in individuals with temporomandibular disorders (TMDs) [2], bruxism [3], tension-type headaches [4], Down syndrome [5], motor neuron diseases [6], and different occlusal features [7]; during orthodontic treatment [8]; and in healthy populations [9]. Moreover, the sEMG measurement is also used to assess the effectiveness of therapeutic methods within the stomatognathic system [10,11,12,13,14]. A decrease in masticatory muscle electromyographic values can indicate the efficacy of botulinum toxin in patients with tension-type headaches [10]. In addition, a significant increase in root mean square (RMS) values within masticatory muscles during maximum voluntary clenching was demonstrated after the compression technique in patients with masseter muscle pain [12]. Thus, sEMG measurements may be an effective means of detecting functional improvements and monitoring the effects of treatment. However, the analysis and interpretation of electromyographic data are complicated and require the use of specialized indices to maximize the reliability, sensitivity, and validity of sEMG measurements. More precisely, to compare the sEMG activity in the field of dentistry, asymmetry (AsI) and activity (AcI) indices [15], maximum voluntary contraction (MVC) [9], percentage overlapping coefficient (POC) [7], torque coefficient [16], antero-posterior coefficient (APC) [17], and total activity (IMPACT) [9] are mainly used. However, several studies also used raw sEMG data or frequency domains [18,19,20,21]. Standardized sEMG values are repeatable, allowing for the assessment and comparison of results among individuals, e.g., between patients and healthy subjects [17,22]. The indices mentioned above are mostly based on the values of the RMS signal amplitude. The sEMG measurements within masticatory muscles usually concern temporalis anterior (TA), superficial masseter (MM), and, less frequently, anterior bellies of the digastric muscle (DA). The activity of the masticatory muscle is assessed during rest, teeth clenching (with and without cotton rolls between teeth), chewing, swallowing, and various movement tasks (mouth opening, protrusion, lateral excursion) [1]. 

The sEMG activity values and symmetry ratio between the right and left side may suggest structural or functional disorders within the stomatognathic system. Changes in the above variables may occur, for example, in the case of a pain response [23], occlusion parameters [24], or abnormal activity patterns in chronic TMDs [25]. Therefore, activity indices are proposed to assess the involvement of the masseter and temporalis muscles during clenching activities. The activity index represents the ratio of one muscle group to the other. AcI values are set between −100 and +100. The negative (−) values indicate the predominance of the temporalis anterior. On the other hand, positive (+) values suggest a masseter muscle advantage. Symmetry evaluation of masticatory muscles is performed using asymmetry indices, which vary between −100 and +100. The negative (−) values indicate the predominance of the left muscle activity. The positive (+) values suggest muscle advantage within the right side of the craniofacial complex. Both indices reaching values close to 0 indicate a symmetrical and equal bioelectric involvement of the masseter and temporalis anterior. The following formulas have been proposed for the assessment of symmetry and activity, according to Naeije et al. and Ferrairo et al. [15,26]:

Activity index (AcI) = (RMS_MM_ − RMS_TA_)/(RMS_MM_ + RMS_TA_) × 100

Asymmetry index (AsI) = (RMS_R_ − RMS_L_)/(RMS_R_ + RMS_L_) × 100

Despite the frequent use of the AsI and AcI indices in research and clinical practice, both indicators are based solely on the masticatory muscle activity during teeth clenching. Hence, the comparison of these indices between individuals may depend on many factors. Thus, reduced reliability of the indices may result from the clenching force generation between subjects, e.g., males vs. females [27], athletes vs. physically inactive individuals [28], long-face individuals vs. individuals with normal facial dimensions [29]. Therefore, we suggest new functional indices for assessing activity and asymmetry within masticatory muscles, taking into account both resting and functional activity. Moreover, we propose a novel Functional Opening Symmetry Index (FOSI) for the digastric muscle. Thus, the presented study aims to analyze novel functional indices of masticatory muscle activity and compare them to existing indices. The electromyographic analysis is based on the sEMG data of patients with TMDs and healthy adults. We hypothesize that the values of novel functional indices will differ between the studied groups, adding a new tool to the electromyographic diagnostics of masticatory muscle imbalance. 

## 2. Materials and Methods

### 2.1. Study Population

The study was carried out according to the Helsinki Declaration’s recommendations and with the consent of the Bioethics Committee of the Medical University of Lublin (KE-0254/331/2015). All participants were informed about the study’s objectives and were aware of the possibility of resigning at any time. Written informed consent was obtained from all subjects involved in the study to publish the presented paper. All tests were performed in the Dentistry Institute (Medical University of Lublin, Lublin, Poland), from October 2016 to the end of June 2019, by experienced researchers. All participants were examined clinically by the same experienced dentist according to the Polish version of a Dual-Axis System of Research Diagnostic Criteria for Temporomandibular Disorders (RDC/TMD) [30]. The inclusion criteria used in the presented study were: female gender, age range 18–30 years, good/very good health status according to the RDC/TMD questionnaire, a full dental arch, the symptoms of TMDs based on an RDC/TMD examination, and compatible occlusal and skeletal classes. The following exclusion criteria were used: periodontal pathology, skin diseases in the head and neck area, neurological disorders in the head and neck area, neoplastic diseases (regardless of type and location), head and neck injuries within the last six months before the examination, surgical treatment in the head and neck area during the previous six months before the test, class II and III according to Angle’s classification, open bite, having an orthodontic appliance, botox therapy, possession of dental prostheses (regardless of type), pregnancy, and psychological disorders [31]. After applying the above criteria, 36 female patients (mean age 23.4 ± 2.6 years) qualified for the TMD group (Table 1). The control group consisted of 42 age-matched women (mean age: 22.4 ± 2.3 years). 

### 2.2. Electromyographic Measurements

The sEMG examination was carried out in a dental chair in a sitting position, with the body perpendicular to the ground, the head resting on the chair’s headrest, and the lower limbs upright and arranged parallel to each other. The headrest’s height was adjusted individually to set the head, neck, and torso of the participants in a straight line. Before electrode placement, the skin was cleansed with a 90% alcohol solution. Ag/AgCl electrodes (SORIMEX, Toruń, Poland) with a diameter of 30 mm and a conductive surface of 16 mm were placed following the Surface Electromyography for Non-invasive Assessment of Muscles (SENIAM) standards [32]. The arrangement of electrodes was carried out by an experienced physiotherapist (the author M.G.), following the course of the muscle fibers of TA, MM and DA muscles (Ueda et al. protocol) [33]. The reference electrode was placed on the forehead. All sEMG examinations were conducted between 8 and 12 a.m. using the 8-channel electromyograph BioEMG III and BioPAK Measurement System (BioResearch Associates, Inc. Milwaukee, WI, USA). Electromyographic activity was recorded in three conditions: at rest (10 s), during maximum voluntary clenching at the intercuspal position (as hard as possible; 3 × 3 s, 2 s rest), and during maximum mouth opening (as wide as possible; 3 × 3 s, 2 s rest) [34]. The electromyographic signals were amplified and purified from 99% of the noise scale on a linear scale using the BioPak digital Noise Buster filter. The post-processing of the sEMG raw signal by the RMS calculation gave average measurement values.

### 2.3. Statistical Calculations

The following formulas were used for the assessment of mean bioelectric activity, AcI, and AsI from the average temporalis anterior, masseter, and digastric muscle RMS potentials recorded during each test, according to Naeije et al. and Ferrairo et al. [15,26]:

Mean bioelectric activity of temporalis anterior (TA) = (TA_R_ + TA_L_)/2

Mean bioelectric activity of masseter (MM) = (MM_R_ + MM_L_)/2

Mean bioelectric activity of digastric (DA) = (DA_R_ + DA_L_)/2

Activity index right-sided (AcI_R_) = (MM_R_ − TA_R_)/(MM_R_ + TA_R_) × 100

Activity index left-sided (AcI_L_) = (MM_L_ − TA_L_)/(MM_L_ + TA_L_) × 100

Activity index both-sided (AcI_tot_) = (MM_R_ + MM_L_ − TA_R_ − TA_L_)/(MM_R_ + MM_L_ + TA_R_ + TA_L_) × 100

Temporalis anterior asymmetry index (AsI_TA_) = (TA_R_ − TA_L_)/(TA_R_ + TA_L_) × 100

Masseter asymmetry index (AsI_MM_) = (MM_R_ − MM_L_)/(MM_R_ + MM_L_) × 100

Digastric asymmetry index (AsI_DA_) = (DA_R_ − DA_L_)/(DA_R_ + DA_L_) × 100

Functional Clenching (FCI) and Functional Opening (FOI) indices were obtained as the ratio of the difference between the mean muscle RMS potentials during activity, including clenching (CL) and opening (MMO), and the mean resting (REST) potentials, using the following formulas:

Functional Clenching Index for TA right-sided (FCI_TA-R_) = CL_TA-R_/REST_TA-R_

Functional Clenching Index for TA left-sided (FCI_TA-L_) = CL_TA-L_/REST_TA-L_

Functional Clenching Index for TA both-sided (FCI_TA_) = (CL_TA-R_ + CL_TA-L_)/(REST_TA-R_ + REST_TA-L_)

Functional Clenching Index for MM right-sided (FCI_MM-R_) = CL_MM-R_/REST_MM-R_

Functional Clenching Index for MM left-sided (FCI_MM-L_) = CL_MM-L_/REST_MM-L_

Functional Clenching Index for MM both-sided (FCI_MM_) = (CL_MM-R_ + CL_MM-L_)/(REST_MM-R_ + REST_MM-L_)

Functional Opening Index right-sided (FOI_R_) = MMO_DA-R_/REST_DA-R_

Functional Opening Index left-sided (FOI_L_) = MMO_DA-L_/REST_DA-L_

Functional Opening Index both-sided (FOI) = (MMO_DA-R_ + MMO_DA-L_)/(REST_DA-R_ + REST_DA-L_)

Next, based on FCI and FOI indices, the following formulas were used for the assessment Functional Clenching Activity Index (FCAI), Functional Clenching Symmetry Index (FCSI), and Functional Opening Symmetry Index (FOSI):

Functional Clenching Activity Index right-sided (FCAI_R_) = (FCI_MM-R_ − FCI_TA-R_)/(FCI_MM-R_ + FCI_TA-R_) × 100

Functional Clenching Activity Index left-sided (FCAI_L_) = (FCI_MM-L_ − FCI_TA-L_)/(FCI_MM-L_ + FCI_TA-L_) × 100

Functional Clenching Activity Index both-sided (FCAI) = (FCI_MM_ − FCI_TA_)/(FCI_MM_ + FCI_TA_) × 100

Functional Clenching Symmetry Index for TA (FCSI_TA_) = (FCI_TA-R_ − FCI_TA-L_)/(FCI_TA-R_ + FCI_TA-L_) × 100

Functional Clenching Symmetry Index for MM (FCSI_MM_) = (FCI_MM-R_ − FCI_MM-L_)/(FCI_MM-R_ + FCI_MM-L_) × 100

Functional Opening Symmetry Index (FOSI) = (FOI_R_ − FOI_L_)/(FOI_R_ + FOI_L_) × 100

The checklist developed by the Strengthening the Reporting of Observational Studies in Epidemiology (STROBE) initiative was used to assess the methodological quality of the presented study [35]. IBM SPSS Statistics 13.3 software was used for statistical analysis. First, the normality of the distribution of variables was verified using the Shapiro–Wilk test and the Kolmogorov–Smirnov test (with Lillierfors correction). Depending on the distribution, the Student *t*-test (T) or Mann–Whitney U test (Z) was used. The significance level was set at 0.05.

## 3. Results

### 3.1. Groups Overview

There were no significant differences in the number of participants (*p* = 1) and age (*p* = 0.26) between the TMD group and controls. However, statistical analysis showed considerable difference within the range of motion (ROM) during maximum active mouth opening between the two groups (44.25 mm vs. 51.52 mm, *p* = 0.00), as presented in Table 1. 

### 3.2. Mean sEMG Activity, and AcI and AsI Indices

Based on statistical analysis, significantly higher values of TA and MM resting activity were observed within all measurements (right-sided, left-sided, both-sided) among TMD patients compared to controls, as presented in Table 2. The differences between groups concerning the mean bioelectrical activity of the DA muscles during resting mandibular position and all examined muscles during clenching and opening tasks were not statistically significant (*p* > 0.05). Moreover, the analysis showed significant differences within AcI_L_ and AcI_tot_ between TMDs and healthy women at rest (*p* = 0.01 and *p* = 0.02, respectively) (Table 2).

The significant difference within asymmetry indices between TMDs and controls was observed only for the AsI _TA_ (−19.37 vs. −5.37, *p* = 0.00) (Table 3).

### 3.3. Functional Indices

Table 4 demonstrates the significant differences between both groups in terms of all Functional Clenching Indices except FCI_MM-R_. In all analyzed FCI indices, the control group showed higher values compared to the TMDs. Moreover, a significant difference between TMDs and controls was observed within FCAI_L_ (14.56 vs. −0.45, *p* = 0.01) (Table 4). 

The significant difference in Functional Symmetry Indices between TMD patients and healthy controls was observed only for the FCSI_TA_ (10.73 vs. −0.62, *p* = 0.03) (Table 5).

## 4. Discussion

Surface electromyography is one of the objective measurements that occurs in modern evidence-based dentistry practice. The sEMG measurement is widely used in dentistry to assess masticatory muscle activity in several functional and structural dysfunctions and assess the effectiveness of therapeutic methods within the stomatognathic system [1]. However, the analysis and interpretation of electromyographic data are complicated and require specialized indices to maximize the reliability, sensitivity, and validity of sEMG measurements. Direct muscle dysfunction or its incorrect activation pattern due to external factors disrupts both the resting and functional muscle activity. The changes mentioned above in muscle activity can be observed in the example of the integrated pain adaptation model [36], e.g., in the presence of myofascial pain syndrome [37]. More precisely, the presence of myofascial trigger points within masticatory muscles may be associated with increased electromyographic values during the resting mandibular position. In addition, bioelectrical masticatory muscle activity may be reduced during teeth clenching [20,23]. 

Despite the frequent use of RMS sEMG data (µV) during clenching/mouth opening and both the AsI and AcI indices, the above-mentioned variables are based solely on the masticatory muscle activity during functional tasks. Therefore, the comparison of mentioned variables between individuals may depend on several external factors unrelated to the dysfunction within the analyzed muscle, e.g., individual anatomy differences, movement patterns, physical activity level, gender, and/or age [27,28,29]. Hence, the analysis of activity and asymmetry indices only during functional activity (without considering the resting activity) may affect the reliable assessment and subsequent interpretation of the obtained results. Thus, the presented study aimed to analyze novel functional indices of masticatory muscle activity and compare them to existing indices. The electromyographic analysis was based on the sEMG data of patients with TMDs and healthy adults. We suggest novel functional indices for assessing activity and asymmetry within masticatory muscles, taking into account both resting and functional activity. Moreover, we propose a novel Functional Opening Symmetry Index for the digastric muscle. The proposed indices assess an integrated analysis of the muscular imbalance within the masticatory muscles. On this basis, we also suggest new asymmetry and activity indices that take into account both functional and resting measurements, although, in the case of proposed indices values, it is not possible to directly distinguish dysfunctions in the area of resting or functional activity. In our opinion, masticatory muscle dysfunction should be considered a comprehensive disorder. The presented study demonstrates the significant differences between both groups in terms of almost all Functional Clenching Indices. Within all analyzed FCIs, the control group showed higher values compared to the TMDs. This resulted from both an increase in resting activity and a decrease in clenching activity in the TMDs group. However, during the studied muscles’ RMS data analysis, only a difference in functional activity was observed. Based on the above observations, we assume that a decrease in functional indices values will be associated with an abnormal/compensatory pattern of masticatory muscle activity. The above-mentioned decrease may result from either a pain adaptation model or other structural factors, as well as from a direct injury within the analyzed muscle.

Despite significant differences in the methodological assumptions between the AsI/AcI and the FCAI/FCSI indices, the presented study showed similarities in the results obtained from the above-presented indicators. Both analysis methods showed asymmetry within the temporalis anterior activity in the group of TMD patients. Moreover, significant differences between the study groups were observed in terms of AcI_R_/AcI_L_/AcI_tot_ in measurement during rest and within FCAI_L_. Hence, both measurement methods seem to be reliable in assessing symmetry and activity within masticatory muscles. The proposed functional indices allow one to determine the presence of muscle dysfunction while taking into account functional and resting activity. Hence, the use of functional indices may improve the assessment of muscle activity in patients with TMDs. Moreover, a comprehensive examination can significantly affect the therapeutic process, helping assess both the masticatory muscles’ resting activity and contraction. However, to verify and confirm the validity and effectiveness of the use of the functional indices, replication studies should be performed. Thus, we strongly encourage discussion of the proposed indices and evaluation of their usefulness in research and clinical practice. The presented study has several limitations. Firstly, the study sample consists of young adult women. Thus, future research should include the male population with an expanded age range. Secondly, the electromyographic analysis is based on the sEMG data of patients with TMDs and healthy adults. Hence, the indices’ effectiveness should be confirmed in studies of other dysfunctions, such as tension-type headaches and bruxism.

## 5. Conclusions

Both functional indices and AsI/AcI seem to be reliable in assessing symmetry and activity within masticatory muscles. Further studies should be performed to verify the masticatory muscles assessment’s effectiveness and suitability using functional indices.

## Figures and Tables

**Table 1:** Group overviews.

	TMDs	Controls	Test	Test Result	*p* Value
*n*	36	42	Z	0.00	1.00
Age (years)	23.42 ± 2.61	22.38 ± 2.34	Z	1.13	0.26
Maximum active mouth opening (mm)	44.25 ± 9.34	51.52 ± 6.42	Z	3.68	0.00 *

* Significant differences (*p* < 0.05) between groups in Mann–Whitney U test (Z).

**Table 2:** Comparison of the mean values (±SD) of bioelectric activity of temporalis anterior (TA), masseter muscle (MM), digastric muscle (DA), and activity indices (AcI) between groups.

Measurement	Indice	TMDs	Controls	Test	Test Result	*p* Value
Rest	TA_R_ (µV)	2.29 ± 0.91	1.48 ± 0.58	Z	−4.44	0.00 *
	TA_L_ (µV)	3.62 ± 1.81	1.66 ± 0.62	Z	−5.82	0.00 *
	TA (µV)	2.95 ± 1.17	1.57 ± 0.56	Z	−5.70	0.00 *
	MM_R_ (µV)	2.32 ± 1.25	1.62 ± 0.57	Z	−2.54	0.01 *
	MM_L_ (µV)	2.62 ± 1.30	1.71 ± 0.78	Z	−3.52	0.00 *
	MM (µV)	2.47 ± 1.04	1.66 ± 0.62	Z	−3.98	0.00 *
	DA_R_ (µV)	2.33 ± 1.04	2.11 ± 1.09	Z	−1.57	0.12
	DA_L_ (µV)	2.25 ± 0.74	2.11 ± 1.13	Z	−1.71	0.09
	DA (µV)	2.29 ± 0.80	2.11 ± 1.01	Z	−1.89	0.06
	AcI_R_	−2.39 ± 27.56	4.76 ± 20.84	T	−1.30	0.20
	AcI_L_	−14.56 ± 27.15	0.45 ± 22.85	T	−2.65	0.01 *
	AcI_tot_	−8.81 ± 23.15	2.68 ± 20.75	T	2.31	0.02 *
Maximum voluntary clenching	TA_R_ (µV)	94.14 ± 54.75	116.56 ± 67.42	Z	1.59	0.11
	TA_L_ (µV)	107.25 ± 55.14	132.61 ± 77.75	Z	1.59	0.11
	TA (µV)	100.70 ± 51.37	124.58 ± 70.64	Z	1.64	0.10
	MM_R_ (µV)	114.18 ± 85.03	123.52 ± 78.60	Z	0.83	0.41
	MM_L_ (µV)	107.21 ± 71.61	128.54 ± 84.72	Z	1.24	0.22
	MM (µV)	110.69 ± 75,00	126.03 ± 74,60	Z	1.21	0.22
	AcI_R_	5.00 ± 30.42	0.09 ± 28.46	T	0.74	0.46
	AcI_L_	−4.79 ± 25.37	−2.31 ± 30.18	T	−0.39	0.70
	AcI_tot_	−0.59 ± 24.48	−0.33 ± 26.45	T	0.04	0.96
Maximum active mouth opening	DA_R_ (µV)	81.96 ± 43.06	76.82 ± 39.42	Z	−0.40	0.69
	DA_L_ (µV)	79.58 ± 49.24	73.15 ± 42.98	Z	−0.32	0.75
	DA (µV)	80.77 ± 44.32	74.99 ± 37.80	Z	−0.43	0.67

* Significant differences (*p* < 0.05) between groups in Student *t*-test (T) or Mann–Whitney U test (Z).

**Table 3:** Comparison of the mean values (±SD) of asymmetry indices (AsI) of temporalis anterior (TA), masseter muscle (MM), and digastric muscle (DA) between groups.

Measurement	Indice	TMDs	Controls	Test	Test Result	*p* Value
Rest	AsI_TA_	−19.37 ± 19.96	−5.37 ± 12.07	T	3.81	0.00 *
	AsI_MM_	−6.64 ± 25.14	−1.08 ± 13.92	T	1.23	0.22
	AsI_DA_	3.14 ± 16.21	3.55 ± 18.95	T	0.10	0.92
Maximum voluntary clenching	AsI_TA_	2.96 ± 10.99	2.43 ± 7.12	T	0.60	0.55
	AsI_MM_	−1.65 ± 8.74	0.40 ± 12.02	T	−1.16	0.25
Maximum active mouth opening	AsI_DA_	−2.29 ± 8.70	−2.72 ± 9.80	T	0.10	0.92

* Significant differences (*p* < 0.05) between groups in Student *t*-test (T).

**Table 4:** Comparison of the mean values (±SD) of Functional Clenching Indices (FCI), Functional Clenching Activity Indices (FCAI), and Functional Opening Indices (FOI) between groups.

Measurement	Indice	TMDs	Controls	Test	Test Result	*p* Value
Functional activity indices	FCI_TA-R_	44.08 ± 25.27	87.86 ± 65.43	Z	4.21	0.00 *
	FCI_TA-L_	34.20 ± 18.23	90.30 ± 67.83	Z	5.29	0.00 *
	FCI_TA_	37.04 ± 16.91	87.79 ± 62.73	Z	5.22	0.00 *
	FCI_MM-R_	66.10 ± 61.23	86.67 ± 65.00	Z	1.78	0.08
	FCI_MM-L_	48.68 ± 27.73	92.57 ± 73.79	Z	3.18	0.00 *
	FCI_MM_	53.56 ± 47.37	87.17 ± 64.42	Z	2.89	0.00 *
	FCAI_R_	7.56 ± 41.19	−4.08 ± 30.80	T	1.43	0.16
	FCAI_L_	14.56 ± 27.15	−0.45 ± 22.85	T	2.65	0.01 *
	FCAI	7.53 ± 32.37	−2.42 ± 31.18	T	−1.38	0.17
	FOI_R_	39.80 ± 24.70	44.47 ± 32.11	Z	0.36	0.72
	FOI_L_	39.85 ± 30.54	44.20 ± 34.69	Z	0.54	0.59
	FOI	38.86 ± 24.96	43.83 ± 30.90	Z	0.47	0.64

* Significant differences (*p* < 0.05) between groups in Mann–Whitney U test (Z) or in Student *t*-test (T).

**Table 5:** Comparison of the mean values (±SD) of Functional Clenching Symmetry Indices (FCSI) and Functional Opening Symmetry Index (FOSI) between groups.

Measurement	Indice	TMDs	Controls	Test	Test Result	*p* Value
	FCSI_TA_	10.73 ± 25.51	−0.62 ± 19.49	T	−2.23	0.03 *
Functional symmetry indices	FCSI_MM_	3.50 ± 40.73	−4.09 ± 33.75	T	−0.90	0.37
	FOSI	2.56 ± 20.64	3.10 ± 22.13	T	0.11	0.91

* Significant differences (*p* < 0.05) between groups in Student *t*-test (T).

## Data Availability

The data presented in this study are available on request from the corresponding author.
